# Integration of onchocerciasis morbidity management and disability prevention services in the healthcare system in Tanzania: a call for action and recommendations

**DOI:** 10.1186/s40794-023-00211-y

**Published:** 2024-01-02

**Authors:** Vivian Mushi, Bruno P. Mmbando, Robert Colebunders

**Affiliations:** 1 Department of Parasitology and Medical Entomology, School of Public Health and Social Sciences, Muhimbili University of Health and Allied Sciences, Dar es Salaam, Tanzania; 2 Department of Zoology and Wildlife Conservation, College of Natural and Applied Sciences, University of Dar es Salaam, Dar es Salaam, Tanzania; 3 National Institute for Medical Research, Tanga Research Centre, Tanga, Tanzania; 4 Global Health Institute, University of Antwerp, Antwerp, Belgium

**Keywords:** Onchocerciasis, Morbidity Management and Disability Prevention, Epilepsy, Ivermectin and Tanzania

## Abstract

Onchocerciasis is among the Neglected Tropical Diseases (NTDs) responsible for dermatological, ophthalmological, and neurological manifestations. With the ongoing burden of onchocerciasis clinical manifestations, morbidity management, and disability prevention services are required to alleviate the suffering of the affected populations. Unfortunately, despite the ongoing transmission of onchocerciasis, morbidity management, and disability prevention services are limited in Tanzania. Therefore, this article highlights the concept of onchocerciasis morbidity management and disability prevention, along with the significance of its adoption in the healthcare system in Tanzania. We further provide recommendations on where and how to start.

## Introduction

**Fig. 1:**
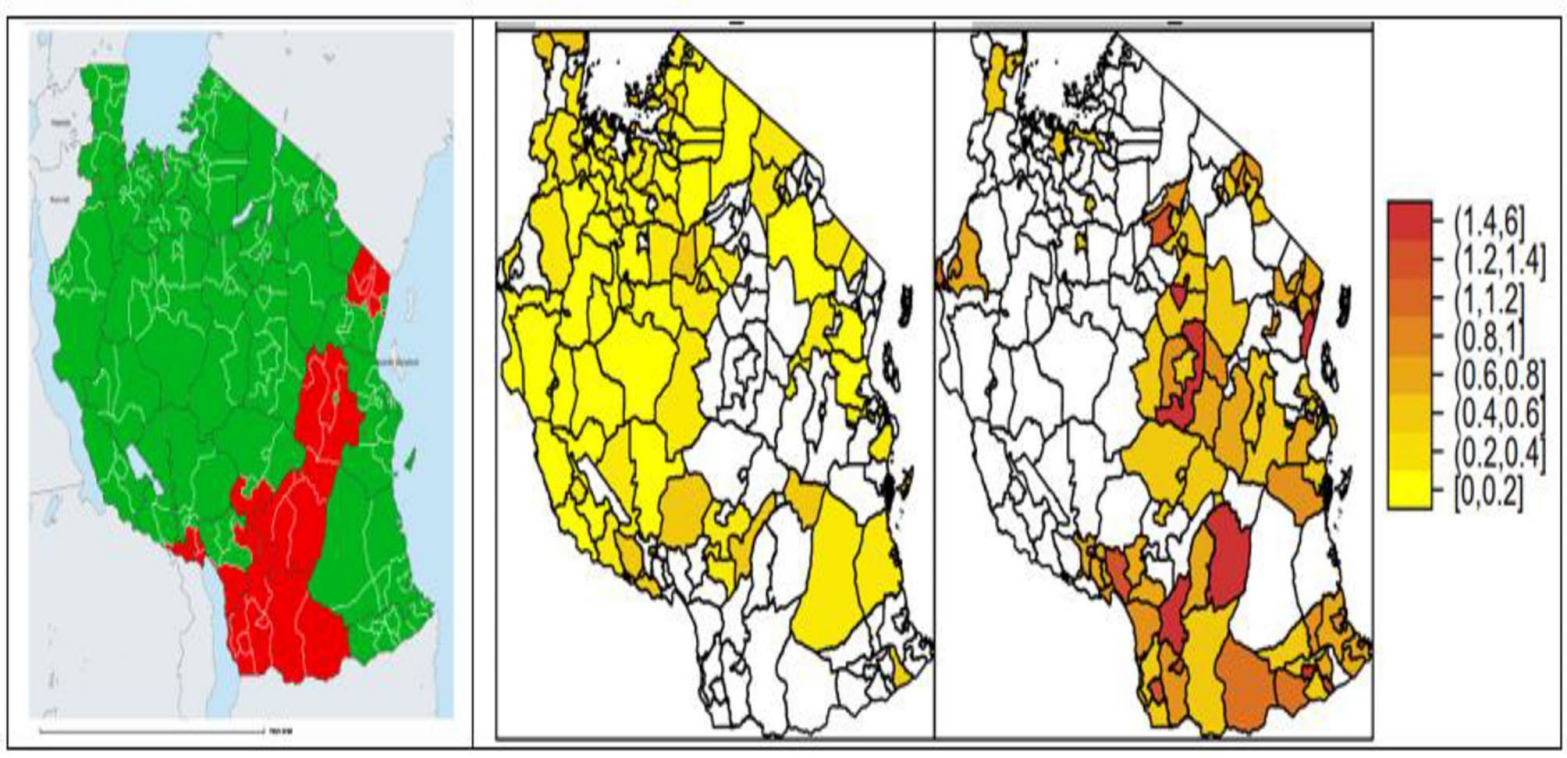
Map of Tanzania showing seven regions (red belt) endemic for onchocerciasis (left), regions with low intensity of epilepsy (middle), and regions with higher intensity of epilepsy (right). Source: ESPEN/WHO and DHIS-2 data

Onchocerciasis is still a public health problem in Tanzania, with more than seven million people at risk of infection in 28 districts in seven regions (Fig. 1) [1]. The disease is responsible for dermatological (skin itching, skin lesions, and onchocercomas), ophthalmological (ocular lesions and blindness), and neurological (onchocerciasis-associated epilepsy [OAE]) manifestations [2, 3]. Baseline mapping of onchocerciasis, first conducted in Tanzania in the 1990s, revealed that the Mahenge area had the highest prevalence of onchocerciasis by nodule palpation (95%) and by skin snip testing (60%) [4–6]. In 1997, Community-Directed Treatment with Ivermectin (CDTI) was started with the help of the African Programme for Onchocerciasis Control (APOC) to reduce *Onchocerca volvulus* (*O. volvulus*) transmission [1, 4, 7]. Ivermectin should be distributed once or twice per year for 16 to 18 years under an optimal coverage of 80% of the total population to eliminate onchocerciasis [8]. From 1997 to 2021 in Tanzania, the overall coverage of ivermectin for the control of onchocerciasis ranged from 39 to 85% of the eligible population [1]. In addition, between 2003 and 2005 larviciding of rivers was used in Tukuyu to reduce blackfly biting rates [9, 10].

Studies conducted in 2018–2019 in the Mahenge onchocerciasis endemic area reported persistent transmission of *O. volvulus* and a high burden of epilepsy [5, 11, 12]. Despite two decades of CDTI, the OV16 antibody seroprevalence among children aged 6–11 years ranged between 2.9 and 40%, and the OAE incidence rate was found to be 131 cases per 100,000 person-years [5, 11, 12]. High OAE incidence and ongoing *O. volvulus* transmission have been linked to suboptimal uptake of ivermectin treatment [13–15]. Therefore, bi-annual CDTI was introduced in Mahenge in 2019, which was recently shown to reduce the incidence of epilepsy, including nodding syndrome [16]. However, even with a decreased OAE incidence by strengthening the onchocerciasis elimination programme, OAE will remain prevalent for many years in the affected communities. Today, a plan to manage onchocerciasis-related morbidities (alleviating the suffering of the affected population) is lacking. As onchocerciasis morbidities are linked with poverty, stigma, and negative psycho-social consequences, there is an urgent need to develop morbidity management and disability prevention (MMDP) services [17].

## Burden of OAE in other sub-saharan African countries

Globally, it was estimated that at least 381,000 individuals were affected by OAE in onchocerciasis-endemic countries in 2015, with different phenotypic presentations, including nodding and nakalanga syndromes [18]. A high epilepsy prevalence (2–8%) has been reported in meso- and hyper-onchocerciasis-endemic regions [19–26]. In those areas, a very high proportion of persons with epilepsy met the criteria of the OAE case definition used for epidemiological studies [27]; for example, 74% in Mvolo, South Sudan [22], 75% in Mundri, South Sudan [21], 85% in Maridi, South Sudan [28], and 93% in the Mbam valley in Cameroon [24].

## Concept of morbidity management and disability prevention

Morbidity management and disability prevention is not a new concept in the context of Neglected Tropical Diseases (NTD) control. In 1997, the World Health Assembly passed resolution WHA 50.29, aiming to eradicate lymphatic filariasis (LF) including a morbidity control strategy [29]. The MMDP Package for LF includes individual treatment or mass drug administration with ivermectin and albendazole, hydrocele surgery, treatment for episodes of adenolymphangitis, and lymphoedema management [29]. Similarly, onchocercal morbidity includes a range of clinical manifestations, that will persist even after the infection has been cleared (skin depigmentation, blindness, and OAE), which significantly increases the risk of mortality for those who are affected [30]. In the early 1990s, before CDTI, it was estimated that onchocerciasis was responsible for 2.5 million disability-adjusted life years (DALYs) [30]. In 2018, it was estimated that OAE roughly accounted for 13% of all years of life with a disability (YLDs) attributable to onchocerciasis and 10% of all YLDs attributable to epilepsy [18, 30]. The CDTI programme has reduced the number of lost DALYs by 1.5 million, with 60% of those caused by dermatological morbidities and 40% by ophthalmological morbidities [19]. Using optimal onchocerciasis elimination strategies the number of DALYs lost could potentially be reduced to 0.7 million by 2030 [30].

## What should an onchocerciasis MMDP service include, and why are MMDP services crucial?

The World Health Organization (WHO) is aiming to interrupt *O. volvulus* transmission in one or more foci in 34 countries by 2030 [31]. The ongoing CDTI programme has a role in alleviating skin manifestations, halting the progression to blindness, and reducing OAE incidence [32]. Treatment of individuals with ivermectin should be possible at health facilities as part of an onchocerciasis MMDP rather than waiting for the next CDTI round. This will not only reduce onchocerciasis-associated symptoms such as onchocerciasis-induced itching, but it may also reduce the frequency of seizures of persons with OAE [33]. Women who missed ivermectin during CDTI because of pregnancy will be able to receive ivermectin when they come to the clinic to vaccinate their children. Moreover, distributing ivermectin at health facilities will increase CDTI coverage.

In places affected by OAE such as Mahenge, the Ministry of Health should decentralize epilepsy treatment services and organize training of primary health care workers to diagnose and treat epilepsy. Additionally, community healthcare workers and ivermectin community drug distributors should be trained to identify persons suspected to have epilepsy and advise them to seek care in an epilepsy treatment center. Integrating MMDP into Tanzania’s healthcare system will improve physical, mental, and social well-being and enhance the quality of life of the affected individuals. A health education programme should raise community awareness about OAE and should be used to explain that OAE can be prevented by the intake of ivermectin. Such a programme should de-stigmatize epilepsy in highly affected areas and increase the schooling of children with epilepsy. Guardians and caregivers of individuals living with OAE should be trained in epilepsy self-care. Finally, advocacy will be needed to convince stakeholders at the national level to promote and implement MMDP services in onchocerciasis endemic areas.

## Challenges to organize MMDP services for onchocerciasis

MMDP services have been developed for LF because specialized services for the management of lymphedema and hydrocoele did not exist in the rural areas where LF is endemic. For onchocerciasis, the context is different since onchocerciasis dermatological manifestations are sequelae that do not need specific treatment, and ophthalmological manifestations have decreased thanks to CDTI. Onchocerciasis-associated epilepsy is a potentially devastating condition that requires appropriate treatment. However, OAE does not present very differently than other forms of epilepsy and therefore, does not require a very different type of treatment. Epilepsy services already exist in the country. However, these services often are not available in the remote affected rural areas, or they work sub-optimally, mainly due to a shortage of trained and motivated staff, interrupted drug supplies, and lack of diagnostic equipment. Therefore, existing epilepsy diagnostic and treatment services need to be strengthened in the affected areas. Affected communities should be involved at every stage of the planning, integration, and implementation of MMDP services. This will raise the acceptability of MMDP services and community members’ sense of programme ownership.

It has also taken a long time before MMDP services were implemented for LF [34]. Only in 2014, Helen Keller International (HKI), with funding from the United States Agency for International Development (USAID), and in collaboration with partners such as the WHO, the Global Alliance to Eliminate Lymphatic Filariasis (GAELF), the Research Triangle Institute (RTI), the African Filariasis Morbidity Project, the Centers for Disease and Prevention (CDC) and universities, was able to implement a project to provide high-quality surgery and disease management services for people suffering from filarial hydrocele and lymphedema, podoconiosis and trachomatous trichiasis [35]. By documenting lessons learned, investigating promising practices, and sharing the knowledge widely, this MMDP project improved data availability and use, filled gaps in the LF knowledge base, contributed to operational research and developed a range of tools and resources for LF MMDP Ministry of Health programmes to implement [34, 35]. This project, initiated by HKI, a non-governmental organization (NGO) with a special interest in supporting projects related to blindness and vision loss and the elimination of diseases of poverty, should be used as an example for initiating a MDDP project for onchocerciasis.

## A road towards integration of MMDP services into Tanzania’s healthcare system: what should be done?

To improve the quality of life of people living with onchocerciasis-associated morbidities, we recommend the following measures:

1. The Tanzania Neglected Tropical Diseases Control Programme (NTDCP) through the Ministry of Health should incorporate onchocerciasis morbidity surveys in the ongoing onchocerciasis transmission assessment surveys. Due to the infrequent assessment of clinical disease in surveillance programmes, there is a paucity of national data on OAE and other clinical manifestations of onchocerciasis. Cost-effectively planning of MMDP services is not possible if areas with high disease burdens are not known. Currently, we know that there is a high OAE burden in Mahenge [16, 33, 36], indicating the need for prioritizing the implementation of MMDP services in this area. In onchocerciasis and LF co-endemic communities, MMDP services should be combined.
2. The use of the District’s Health Information System (DHIS2) database together with the knowledge of the onchocerciasis endemic sites should be used to identify other areas for MMDP prioritizing. Analyzing epilepsy DHIS2 data may reveal other areas in Tanzania with high epilepsy prevalence. Indeed, Tanzania is endemic of *Taenia solium*, with prevalences of infection across the country ranging from 4 to 53.7% using Western Blot Assay and Enzyme-linked Immunoassay [37, 38]. *Taenia solium* infections may cause neurocysticercosis and epileptic seizures [25]. Currently, there is also no specific programme tailored for the management of neurocysticercosis. Therefore, onchocerciasis MMDP services could also be useful for areas with a high prevalence of neurocysticercosis. Combining MDDP services for several NTDs will decrease its cost.
3. Given that ivermectin treatment should be a component of the onchocerciasis MMDP package, the Ministry of Health, through NTDCP, should ensure its accessibility and availability in health facilities. The suboptimal uptake of ivermectin during mass drug administration has been reported in onchocerciasis endemic areas, especially among persons with epilepsy [39]. Persons with epilepsy, in onchocerciasis endemic areas, who failed to take ivermectin during CDTI should be able to obtain ivermectin at health facilities. In addition, an uninterrupted supply of anti-seizure medication should be available at epilepsy treatment clinics.
4. MMDP services should be integrated into the healthcare system. This requires collaboration with various stakeholders to ensure the availability of adequate resources, well-trained healthcare workers, and commodities for the management of MMDP. Therefore, the Ministry of Health (the NTD and the mental health programme) should be open to collaborating with Non-Governmental Organizations (NGOs), policymakers, and donors to support the MMDP activities.

## Conclusions

In conclusion, the integration of MMDP services for several NTDs within the healthcare system targeting high-risk areas should be the preferred strategy to reduce the number of years lost due to NTD-related disabilities and improve the quality of life of people affected and their communities.

## Data Availability

All the data used are from the references provided.

## List of abbreviations

APOC: African Programme for Onchocerciasis Control
CDC: Centers for Disease and Prevention
CDTI: Community Directed Treatment with Ivermectin
DALYs: Disability-Adjusted Life Years
DHIS: Districts Health Information System
GAELF: Global Alliance to Eliminate Lymphatic Filariasis
HKI: Helen Keller International
MMDP: Morbidity Management and Disability Prevention
NGOs: Non Governmental Organizations
NTD: Neglected Tropical Disease
NTDCP: Neglected Tropical Diseases Control Programme
OAE: Onchocerciasis associated Epilepsy
RTI: Research Triangle Institute
USAID: United States Agency for International Development
WHO: World Health Organization
YLDs: Years of Life with a Disability
